# Glyphosate and its formulation Roundup impair pig oocyte maturation

**DOI:** 10.1038/s41598-020-68813-6

**Published:** 2020-07-20

**Authors:** Marcella Spinaci, Chiara Nerozzi, Car lo Tamanini, Diego Bucci, Giovanna Galeati

**Affiliations:** Department of Veterinary Medical Sciences, University of Bologna, Via Tolara di Sopra, 50, 40064 Ozzano dell’Emilia, Bologna, Italy

**Keywords:** Cell biology, Physiology, Environmental sciences

## Abstract

Glyphosate, formulated as glyphosate-based herbicides (GBHs) including the best-known formulation Roundup, is the world's most widely used herbicide. During the last years, the growing and widespread use of GBHs has raised a great concern about the impact of environmental contamination on animal and human health including potential effect on reproductive systems. Using an in vitro model of pig oocyte maturation, we examined the biological impact of both glyphosate and Roundup on female gamete evaluating nuclear maturation, cytoplasmic maturation and developmental competence of oocytes, steroidogenic activity of cumulus cells as well as intracellular levels of glutathione (GSH) and ROS of oocytes. Our results indicate that although exposure to glyphosate and Roundup during in vitro maturation does not affect nuclear maturation and embryo cleavage, it does impair oocyte developmental competence in terms of blastocyst rate and cellularity. Moreover, Roundup at the same glyphosate-equivalent concentrations was shown to be more toxic than pure glyphosate, altering steroidogenesis and increasing oocyte ROS levels, thus confirming that Roundup adjuvants enhance glyphosate toxic effects and/or are biologically active in their side-effect and therefore should be considered and tested as active ingredients.

## Introduction

Glyphosate (Gly), or *N*-(phosphonomethyl)glycine, is a non-selective herbicide widely used worldwide to control weeds^1^. Gly is commonly applied as part of glyphosate-based herbicides (GBHs), which include the popular commercial formulation Roundup (R), in which adjuvants enhance the herbicidal properties.

During the last years, the growing and widespread use of GBHs has raised a great concern about the impact of environmental contamination on animal and human health. Human and animal Gly exposure may occur through various routes such as food and drinking water, skin contact or by inhalation^2,3^. Only a small amount of Gly is metabolized by mammals, while the majority is excreted unmodified by urine in which Gly residues have been detected in both humans^2,4^ and animals as rats^5^, cows^6,7^, rabbits^7^, dogs and cats^8^.

The possible risk associated with Gly exposure to human and animal health is a matter of an intense public debate for both its potential carcinogenic and non-carcinogenic effects, including potential adverse effects on nervous, digestive, endocrine and reproductive systems^9–14^. However, findings of both in vitro and in vivo studies are conflicting and several authors concluded that Gly is safe at levels below regulatory permissible limits^15–22^.

Nevertheless, GBHs have been clearly demonstrated to exert their effects through a chemical endocrine disruption; in fact, they have been shown to impair the androgen/estrogen balance^23,24^, thus determining an endocrine disarray in cell lines (e.g. ^25,26^). Furthermore, R exposure in rats has been demonstrated to interfere with both steroidogenic enzymes and reproductive health^27,28^. It has been suggested that the modification in reproductive hormone concentrations induced by GBHs could be due to changes in the number and activity of Leydig cells and modification of steroidogenic acute regulatory protein (StAR) or aromatase levels and activity^26,29,30^. According to another study, R seems to exert an inhibitory effect at the hypothalamic-pituitary level and to disrupt cyclic adenosine monophosphate (cAMP)/protein kinase A (PKA) pathway and corticosterone synthesis in the adrenal gland^31^. Moreover, Gly and R have been demonstrated to impair bovine and swine granulosa cells growth and steroid production^32–34^. In female rat and mice, Gly and GBHs have been reported to cause hormonal imbalances, oxidative stress and alterations in folliculogenesis including an increase of atretic follicles^35,36^. Recently Yahfoufi et al.^37^ reported that Gly exposure of mature mouse oocyte (MII) induces spindle fibre destruction, disturbance in chromosomal alignment, depletion of intracellular zinc bioavailability and ROS accumulation. Similar effects were found in mouse embryos exposed to Gly during embryo culture^37^. Induction of oxidative stress and apoptosis were also observed in bovine embryos cultured in presence of R^38^.

However, very few data are available in literature on possible effects of Gly and GBHs on mammal oocyte maturation, which prepare oocyte for fertilization events and affect early embryonic development^39^. Zang et al.^40^ observed that Gly interferes with in vitro mouse oocyte maturation impairing nuclear maturation, generating oxidative stress and inducing DNA damage and early apoptosis.

On these bases, the objective of this study was to characterize the impact of Gly and R on female gamete using an “in vitro” model of pig oocyte maturation (IVM) evaluating nuclear maturation, cytoplasmic maturation and developmental competence of oocytes, steroidogenic activity of cumulus cells as well as intracellular levels of glutathione (GSH) and ROS of oocytes. We tested concentrations ranging from either 360 µg/mL Gly or 0.1% Roundup (containing 360 µg/mL Gly) to 70-fold lower on the basis of previous in vitro studies on reproductive tissues and gametes^25,30,33,40,41^.

## Results

### Effect of Gly and R on nuclear and cytoplasmic maturation

When COCs were matured in presence of Gly at 0, 5, 10, 100, 200 and 360 µg/mL or R at the same Gly-equivalent doses, no significant variations in the proportion of oocytes completing nuclear maturation showing a MII nuclear morphology were recorded (Table 1).

**Table 1:** Effect of Gly and Roundup on percentage of oocytes in metaphase II at 44 h of culture. Data represent the mean ± SD of six replicates repeated in different experiments.

	0	5	10	100	200	360
**Gly (µg/mL)**						
MII (%)	91.1 ± 7.1	91.9 ± 6.6	88.7 ± 5.2	88.0 ± 7.8	92.1 ± 5.1	90.9 ± 5.8
Oocytes (n°)	272	266	259	282	259	253
**Roundup (µg/mL Gly eq)**						
MII (%)	92.8 ± 7.6	91.8 ± 4.1	90.9 ± 6.9	90.3 ± 6.8	91.3 ± 6.6	92.8 ± 4.1
Oocytes (n°)	264	239	250	264	255	261

Gly or R addition during oocyte maturation at all the concentrations tested did not influence, after IVF with frozen-thawed semen, the percentage of penetrated oocytes, monospermic oocytes and percentage of penetrated oocytes with at least one male pronucleus (Table 2).

**Table 2:** Effect of Gly and Roundup addition during IVM on fertilization rate, monospermy rate and on the ability of oocytes to sustain male pronucleus formation after in vitro fertilization. Data represent the mean ± SD of six replicates repeated in different experiments.

	0	5	10	100	200	360
**Gly (µg/mL)**						
Penetration rate	75.6 ± 4.0	73.3 ± 5.2	73.5 ± 8.1	78.2 ± 5.8	78.1 ± 5.2	72.7 ± 5.0
Monospermy rate	65.3 ± 12.2	62.8 ± 9.7	51.3 ± 12.6	60.2 ± 15.6	63 .2 ± 13.1	53 .5 ± 6.4
Male pronuclear formation	99.4 ± 1.5	99.4 ± 1.6	98.5 ± 2.4	99.4 ± 1.5	99.3 ± 1.7	98.5 ± 2.5
Oocytes (n°)	284	284	243	221	244	262
**Roundup (µg/mL Gly eq)**						
Penetration rate	76.0 ± 5.2	75.4 ± 4.8	74.5 ± 10.1	75.6 ± 8.7	68.8 ± 10.0	69.0 ± 8.9
Monospermy rate	54.4 ± 11.8	56.5 ± 9.9	63.7 ± 14.2	55.4 ± 11.1	66.9 ± 11.0	56.9 ± 12.5
Male pronuclear formation	100.0 ± 0.0	98.7 ± 2.0	98.8 ± 2.9	97.2 ± 3.3	98.7 ± 2.1	99.5 ± 1.3
Oocytes (n°)	267	227	233	254	228	242

**Figure 1:**
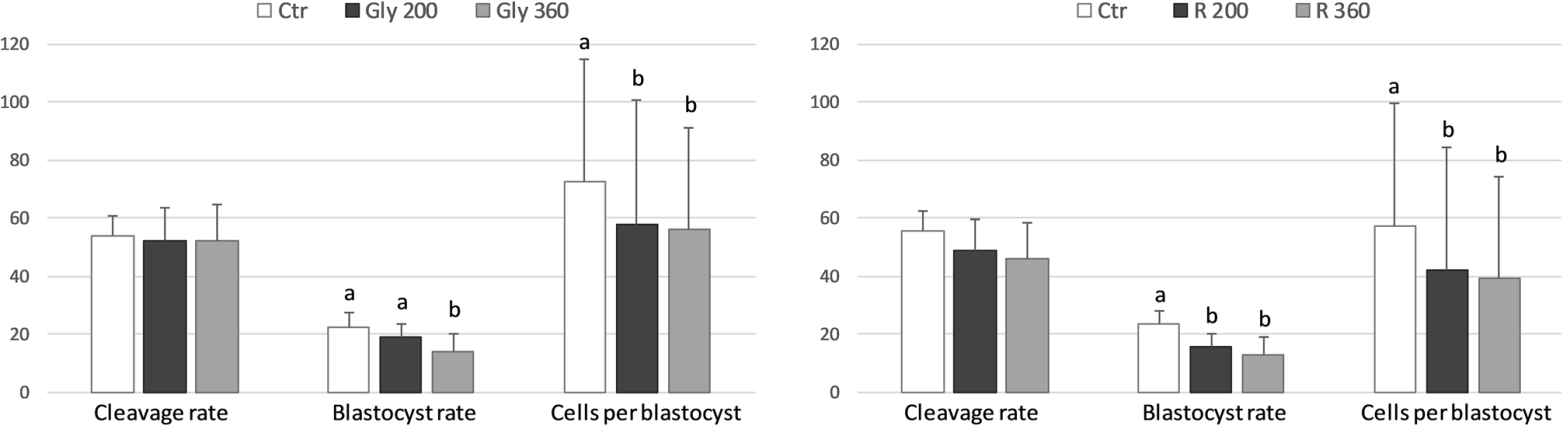
Effect of Gly (left panel) and R (right panel) addition during IVM on cleavage rate, blastocyst rate and blastomere number per blastocyst. Data represent the mean ± SD of five replicates repeated in different experiments. Different letters represent significant difference for P < 0.05 between treatments.

While the presence of Gly during IVM at all the doses tested (0, 200, 360 µg/mL) did not affect the cleavage rate, it caused a significant (p < 0.01) reduction of the percentage of oocytes that developed to blastocyst stage at the higher concentration (360 µg/mL). Moreover, a significant decrease in the mean number of blastomeres per blastocyst was observed starting from Gly 200 µg/mL (p < 0.05) (Fig. 1, left panel).

R did not influence the cleavage rate; however, at all the concentrations tested (200 and 360 µg/mL Gly-equivalent) it induced a significantly lower blastocyst rate (p < 0.05 and p < 0.001 for R 200 and R 360, respectively) and mean number of blastomeres per blastocyst (p < 0.01 and p < 0.001 for R 200 and R 360 respectively) compared to control (Fig. 1, right panel).

### Effect of Gly and R on cumulus cell steroidogenesis

**Figure 2:**
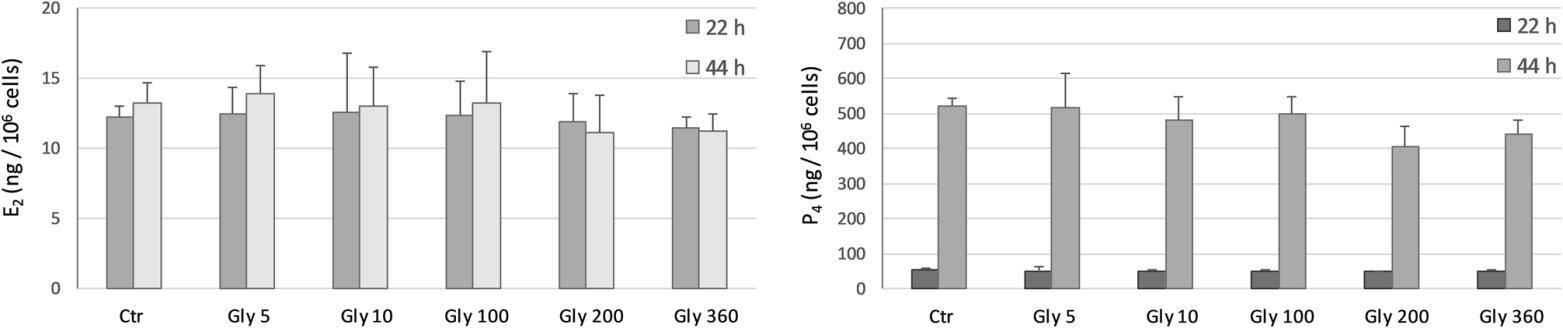
Effect of Gly (0, 5, 10, 100, 200, 360 µg/mL) on E2 (left panel) and P4 (right panel) production by porcine cumulus cells after 22 h and 44 h of culture. Data represent mean ± SD of 4 independent experiments.

**Figure 3:**
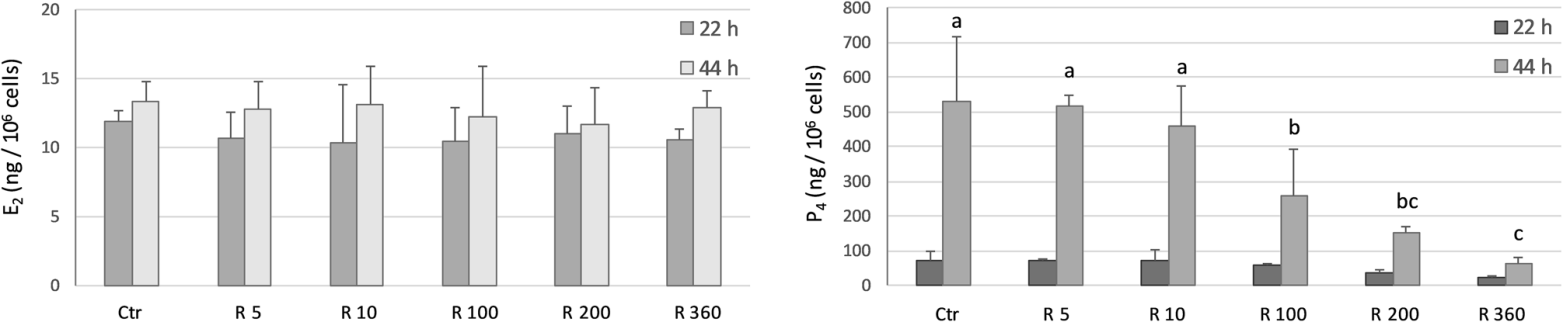
Effect of R (0, 5, 10, 100, 200, 360 µg/mL Gly-equivalent) on E2 (left panel) and P4 (right panel) production by porcine cumulus cells after 22 h and 44 h of culture. Data represent mean ± SD of 4 independent experiments. Different letters on the same bar type represent significant difference for P < 0.01 between treatments.

Basal steroid production by COCs after 22 and 44 h of culture is shown in Figs. 2 and 3.

The production of P4 was significantly higher (p < 0.0001) at 44 h of culture compared to 22 h irrespective of Gly and R concentrations.

E2 and P4 production was not affected by Gly exposure in none of the 2 days of culture (Fig. 2).

None of the R concentrations tested induced any effect on E2 production, in either the first and the second day of culture (Fig. 3, left panel), and on P4 production after 22 h (Fig. 3, right panel).

After 44 h of culture, R inhibited P4 production in a dose dependent manner starting from 100 µg/mL (p < 0.0001) (Fig. 3, right panel).

### Effect of Gly and R on GSH and ROS levels

**Figure 4:**
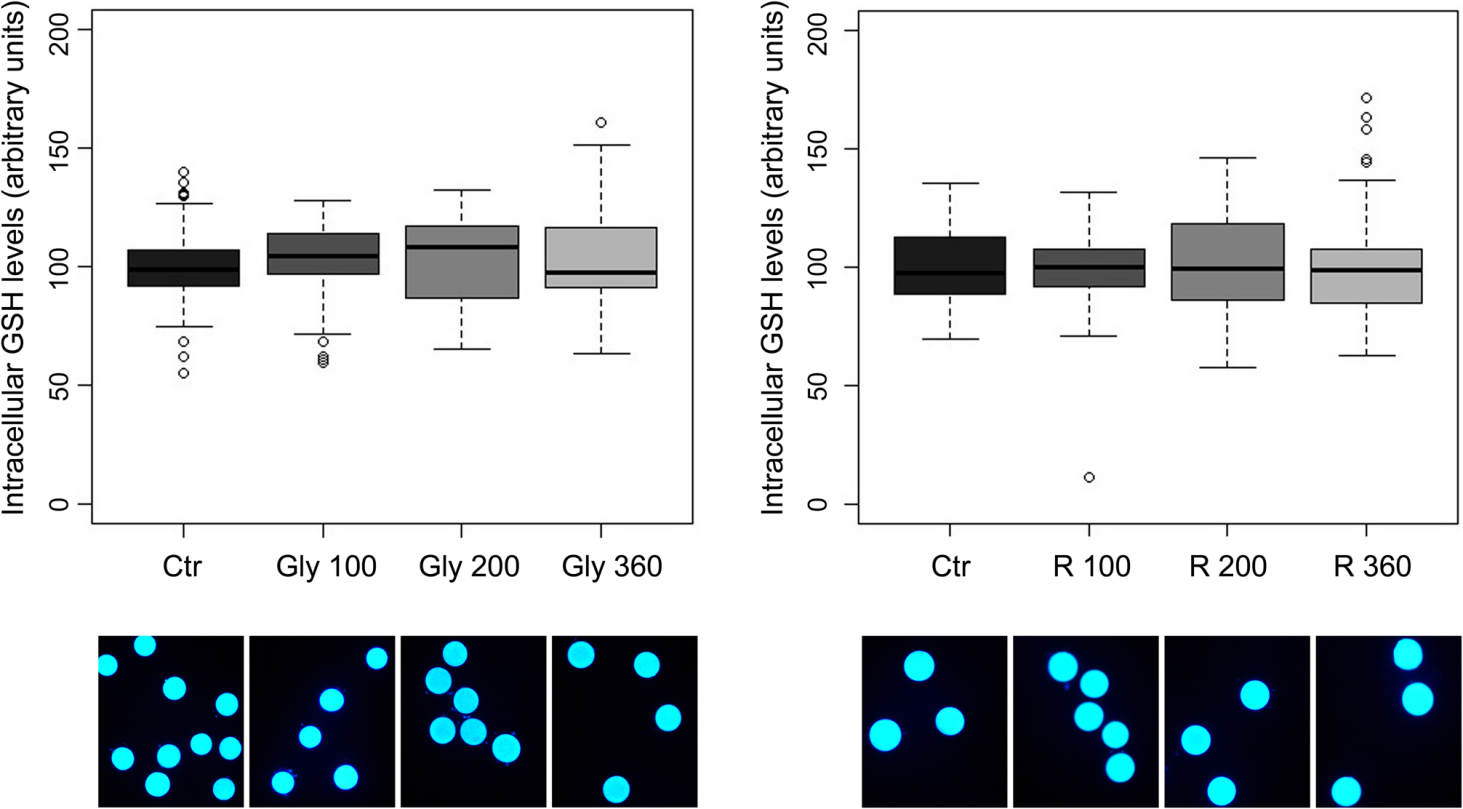
Upper panel. Box plots for intracellular GSH levels of oocytes matured in presence of Gly (left panel) and R (right panel). Oocytes were dyed with CellTracker Blue. Central lines represent median; boxes represent 25–75 percentile; whiskers represent minimum and maximum; dots represent outliers. The experiment was replicated 5 times with 15–20 oocytes each time. Lower panel. Representative epifluorescent microphotographic images of porcine oocytes matured in presence of Gly (left panel) and R (right panel) stained with CellTracker Blue to detect intracellular GSH levels.

The oocyte GSH levels were not statistically influenced by the exposure during IVM to Gly or R at the concentrations of 100, 200 and 360 µg/mL (Fig. 4).

**Figure 5:**
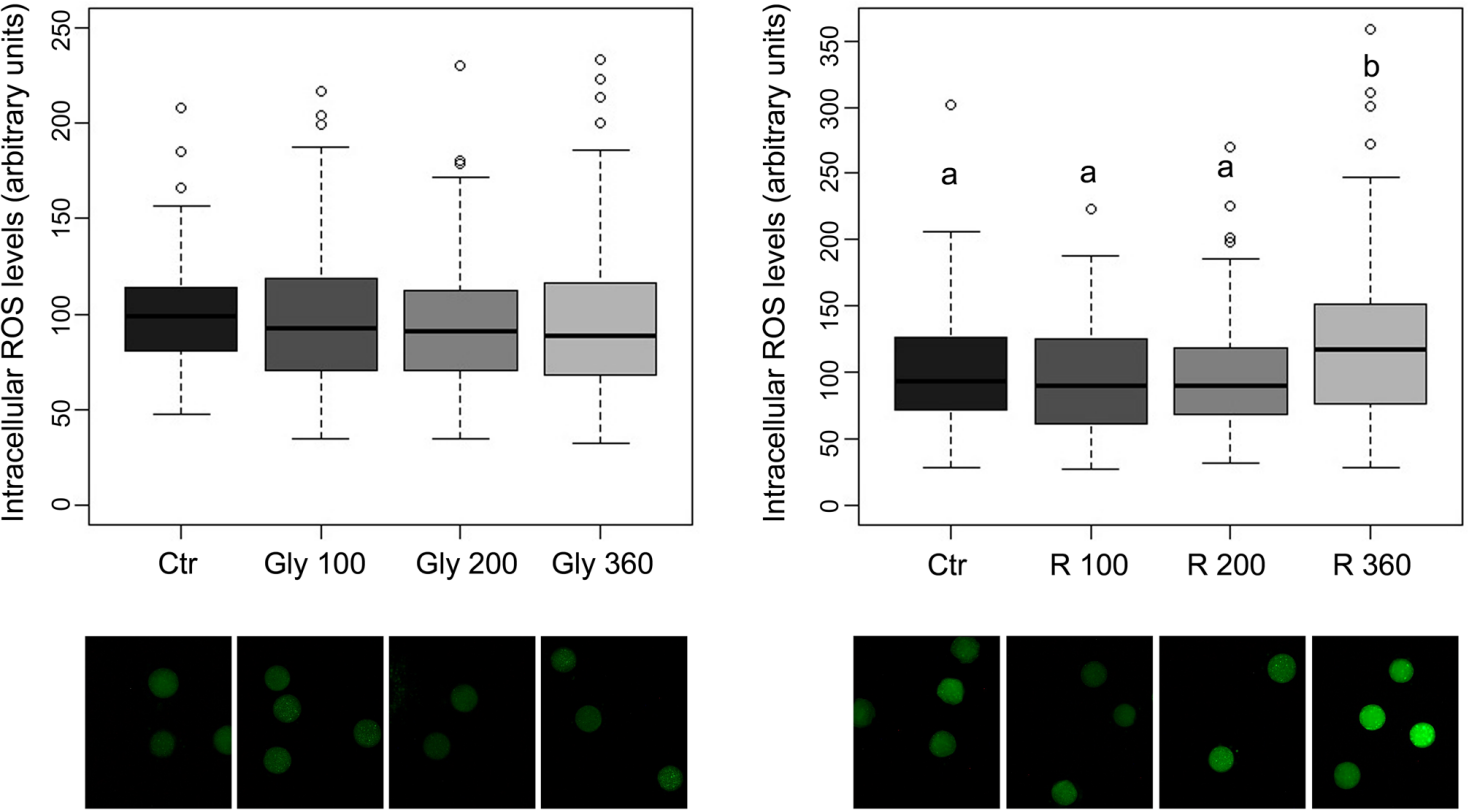
Upper panel. Box plots for intracellular ROS levels of oocytes matured in presence of Gly (left panel) and R (right panel). Oocytes were dyed with H2DCFDA. Central line represents median; boxes represent 25–75 percentile; whiskers represent minimum and maximum; dots represent outliers. Different letters within same graph represent significant difference for P < 0.05 between treatments. The experiment was replicated 5 times with 15–20 oocytes each time. Lower panel. Representative epifluorescent microphotographic images of porcine oocytes matured in presence of Gly (left panel) and R (right panel) stained with H2DCFDA to detect intracellular ROS levels.

While Gly presence during in vitro maturation did not modify intracellular ROS levels (Fig. 5, left panel), R at the highest concentration tested (360 µg/mL Gly-equivalent) significantly increased intracellular ROS levels (p < 0.01) (Fig. 5, right panel).

## Discussion

The purpose of this study was to evaluate the impact of Gly and R exposure on female gamete using an in vitro model of pig oocyte maturation.

Pig is an important species not only for agriculture, but also for research as a biomedical animal model due to anatomical and physiological similarity compared to human^42,43^. Moreover, according to 3Rs principle, the use of female gametes from non rodent-species, such as farm animals, has been considered to serve as useful in vitro screening test for reproductive toxicology^44^.

As a first step of this study we investigated the effect of Gly and R exposure during IVM on nuclear maturation of pig oocytes. None of the concentrations tested (5, 10, 100, 200 and 360 µg/mL Gly or Gly-equivalent doses for R) modified the percentage of oocytes reaching MII stage compared to control. These results are in contrast with the only study performed up to now on the effect of Gly exposure during IVM on nuclear maturation of mammalian oocytes^40^. In that study, 200 and 500 µM Gly (33.8 and 84.5 µg/mL respectively) decreased the proportion of polar body extrusion of mouse oocytes due to misaligned chromosomes and abnormal spindle morphology; the authors suggested that Gly toxicity on mouse oocytes could be mediated by the increase of intracellular ROS levels.

The discrepancy between Zang et al.^40^ and our results could be due to a species-dependent sensitivity to Gly and/or it can be ascribed to different cultural conditions: Zang et al.^40^ matured mouse oocytes in M2 medium, while, in our model, pig IVM was performed in NCSU 37 medium supplemented with cysteine and β-mercaptoethanol, molecules known to reduce pig oocyte ROS levels, and 10% porcine follicular fluid (pFF), endowed with high radical scavenging activity elicited from SOD isoenzymes^45–48^; these supplementations, increasing the antioxidant activity of the system, may have masked the potential Gly toxic effects on nuclear maturation. In fact, it has been recorded that cell damage encompassed by ROS are difficult to detect in pig oocytes cultured in a medium supplemented with 10% pFF, even in the presence of ROS generated by the hypoxanthine-xanthine oxidase system^47^. Anyway, it must be kept in mind that in vivo oocytes are normally protected from the harmful effects of ROS by anti-oxidant enzymes which are present in the follicular fluid^49^ and therefore it can be assumed that our culture system is capable of closely mimicking the in vivo environment during oocyte maturation. In our pig model, a significant increase of the intracellular levels of ROS with R at the concentration of 360 µg/mL, but not with pure Gly, was recorded; this increase, however, was not as dramatic as that recorded in mouse oocyte after Gly exposure^40^.

In order to evaluate the potential toxic effect of Gly and R exposure on cytoplasmic maturation, as a first step, fertilization parameters and oocytes ability to decondense sperm head and sustain male pronucleus formation after in vitro fertilization were evaluated. As observed for nuclear maturation, no detrimental effect of all the Gly and R doses tested on these parameters was recorded. Adequate oocyte GSH levels are needed in order to reduce protamine disulfide bonds that represent the first step in the induction of sperm nuclear decondensation and hence male pronucleus formation after in vitro fertilization^50^. The maintained sperm nuclear decondensing ability of the exposed oocytes agree well with the absence of any effect of either Gly or R on intracellular GSH levels which was recorded in this study. Even if an inverse relationship between oocytes intracellular ROS and GSH levels has been observed by many authors^45–48^, in this study, R 360 significantly increased the intracellular levels of ROS; it is likely that this rise was not so strong to significantly reduce oocyte GSH levels and, in turn, to impair oocyte decondensing activity.

The developmental competence of exposed oocytes after IVF was used as a further parameter of proper cytoplasmic maturation. Even the highest doses tested of both Gly and R had no effect on embryo cleavage. By contrast, R, and to a lesser extent Gly, induced a dose dependent reduction of both blastocyst rate and blastomere number per blastocyst. Therefore, oocyte exposure to R and Gly during IVM impaired the acquisition of a proper cytoplasmic maturation leading to a reduction of developmental competence, even if pesticides were not more present during embryo culture. This toxic effect was more evident with R compared to Gly, thus suggesting a synergistic effect provoked by the adjuvants present in the commercial formulation.

Concerning the effects on cumulus cell steroidogenesis, while Gly did not induce any alteration of E2 and P4 levels, R decreased P4 (but not E2) production. In our system, P4 secretion by COCs dramatically increased during the second half of culture, as already observed^48,51,52^, likely due to cumulus cell differentiation-luteinization and R was markedly effective in inhibiting this P4 increase as P4 produced after 48 h of culture was significantly lower in R treated groups, starting from 100 µg/mL, as compared to control. Similarly, treatment of bovine granulosa cells with Gly (10 and 300 µg/mL) had no effect on P4 and E2 production^33^ while R (10 µg/mL) dramatically decreased steroid levels (P4 and E2). Other researches carried out by Gigante et al.^34^ on swine granulosa cell showed that Gly (0.2, 4 and 16 μg/mL) induced a significant inhibitory effect on granulosa cell E2 secretion, viability and proliferation; in contrast, P4 secretion was stimulated at all tested concentrations. It must be considered that the researches carried out by Perego et al.^33^ and Gigante et al.^34^ were performed on plated granulosa cell with the addition of testosterone or androstenedione as estradiol precursors while our model, consisting of cumulus cell-oocyte complexes, is completely different. In fact, cumulus cells and mural granulosa cells are phenotypically/functionally different: cumulus cells play an essential role in the normal growth and development of the oocyte, while mural granulosa cells primarily exert an endocrine function and support follicle growth^53^. Moreover, evidence exists that oocyte plays an active role in determining the fate of follicle somatic cells; results obtained by Coskun et al.^54^ demonstrated that porcine oocytes secrete molecule(s) that inhibits steroid production by cumulus and granulosa cells. Therefore, based on these information we cannot strictly compare our results on steroidogenesis with those obtained by Perego et al.^33^ and Gigante et al.^34^.

P4 produced by cumulus complexes has been reported to positively influence porcine oocyte cytoplasmic maturation and to improve developmental competence to the blastocyst stage following IVF^55,56^. Therefore, the more serious negative effects of R compared to the pure molecule on oocyte developmental competence can possibly be due, at least in part, to the observed perturbation induced by R on P4 production by cumulus cells. Anyway, the mechanism through which R exerts its effects on cumulus cell steroidogenesis needs further investigations.

The decrease in P4 production by cumulus cells recorded in presence of R could have contributed to the increase in intracellular ROS levels induced by R 360. Previous studies, in fact, demonstrated that progesterone possesses antioxidant properties that are blocked by RU486, a P4 receptor antagonist^57^.

All these results support the hypothesis that surfactants/adjuvants present in GBHs are responsible for the increased toxicity of Gly^30,58^ or exert an intrinsic toxicity inducing membrane disruption^30,59^, apoptosis^60^, inhibition of mitochondrial respiration^30,59,61^ and DNA damage^62^.

To the best of our knowledge, the present study is the first work describing the effects of Gly and R on pig oocytes maturation.

Our results indicate that the exposure to Gly and its commercial formulation R during IVM, even if it does not affect nuclear maturation and embryo cleavage, impairs oocyte developmental competence in term of blastocyst rate and cellularity. Moreover, R at the same Gly-equivalent concentrations resulted to be more toxic than pure Gly, altering steroidogenesis and increasing oocyte ROS levels, thus confirming that R adjuvants enhance Gly toxic effects and/or are biologically active in their side-effect and therefore should be considered and tested as active ingredients.

Glyphosate concentrations detected in human urine has been reported to be at ng/mL levels with higher level in specifically exposed individuals^22,63–65^; mean levels of 0.26 µg/mL (range < 0.020–17.2 µg/mL) in occupationally exposed workers have been recently reported^66^. These concentrations are far lower than those observed to be toxic in this study. Blood glyphosate levels recorded in human acute intoxications were 61 µg/mL (range 0.6–150 µg/mL) and 838 µg/mL respectively in mild–moderate and severe intoxication cases^67^, concentrations of the order of magnitude of those that were toxic in our study.

Obviously, the effects induced by Gly and formulants should be lower in vivo than in culture, and in vitro methods cannot provide the information that can be derived from in vivo tests. Nevertheless, in vitro maturation of pig oocytes, that can be obtained in large number from ovaries collected at the slaughterhouse, can be used as a reliable model to screen toxic agent for female gamete allowing the reduction of the number of laboratory animals used in vivo accordingly to 3R principles.

The consequences of the massive use of GBHs remain a matter of concern on public health. We found that Gly and R exposure during IVM detrimentally affect the subsequent developmental ability of embryos, providing further evidence of their potential toxic effect on female reproductive system that is worth of a deeper investigation.

## Methods

### Chemicals

*N*-(Phosphonomethyl)glycine (Glyphosate, Gly, CAS Number 1071-83-6) as well as the other chemicals, unless otherwise specified, were purchased from Sigma-Aldrich (Saint-Louis, MO, USA) except Roundup Bioflow (Roundup Bioflow, Monsanto Europe N.V., Anversa, Belgium) containing 360 g/L of glyphosate acid in the form of 480 g/L isopropylamine salts of glyphosate (41.5%), water (42.5%) and surfactant (16%; chemical name, CAS number and/or exact percentage have been withheld as a trade secret).

### Oocytes collection and in vitro maturation (IVM)

Ovaries were collected from pre‐pubertal gilts at a local slaughterhouse and transported (in 0.9% wt/vol NaCl solution) to the laboratory within 2 h. Cumulus‐oocyte complexes (COCs) were aspirated from antral follicles, 3–6 mm in diameter, with a 18‐gauge needle fixed to a 10‐mL disposable syringe. Intact COCs were selected under a stereomicroscope and only COCs with more than three layers of compact cumulus cells and with uniform cytoplasm were transferred into a petri dish (35 mm, Nunclon, Denmark) prefilled with 2 mL of modified PBS supplemented with 0.4% BSA. After three washes in NCSU 37^68^ supplemented with 5 μg/mL insulin, 1 mM glutamine, 0.57 mM cysteine, 10 ng/mL epidermal growth factor (EGF), 50 μM β-mercaptoethanol and 10% porcine follicular fluid (IVM medium), groups of 50 COCs were transferred to a Nunc 4-well multidish containing 500 μL of the same medium per well and cultured at 39 °C in a humidified atmosphere of 5% CO_2_ in air. For the first 22 h of in vitro maturation the medium was supplemented with 1.0 mM db-cAMP and 0.12 IU/mL Pluset (Carlier, Italy). For the last 22 h COCs were transferred to fresh maturation medium^69^.

### Evaluation of nuclear maturation

In order to assess the effect of Gly and R on nuclear maturation, pig COCs were exposed during in vitro maturation period (44 h) to 0, 5, 10, 100, 200 and 360 µg/mL Gly or R at the same Gly-equivalent doses.

At the end of the maturation period the oocytes were denuded by gentle repeated pipetting and then mounted on microscope slides, fixed in acetic acid/ ethanol (1:3) for 24 h and then stained with Lacmoid. The oocytes were observed under a phase contrast microscope in order to evaluate the meiotic stage achieved (total number of oocytes examined 3,124). Oocytes with a nuclear morphology corresponding to metaphase-II stage (MII) were considered mature^70^.

### Evaluation of cytoplasmic maturation

Cytoplasmic maturation was assessed by evaluating:

(*a*)* insemination parameters and ability of oocytes to sustain male pronucleus formation after in vitro fertilization.*

At the end of the maturation period in presence of Gly (0, 5, 10, 100, 200 and 360 µg/mL) or R at the same Gly-equivalent doses, the oocytes were fertilized with frozen boar semen purchased from a commercial company (Inseme S.P.A., Modena, Italy). Straws were thawed in a waterbath at 37 °C under agitation for 30 s and immediately diluted, at the same temperature, in Beltsville Thawing Solution (BTS) at a dilution rate 1:3.

After 1 h semen was washed twice with BTS and finally resuspended with Brackett and Oliphant’s medium^71^ supplemented with 12% fetal calf serum (Gibco, Invitrogen, Italy) and 0.7 mg/mL caffeine (IVF medium). Forty-five to fifty oocytes freed from cumulus cells were washed twice in IVF medium and transferred to 500 µL of the same medium containing 0.25 × 10^6^ sperm/mL. After 1 h of gamete coincubation, oocytes were transferred to fresh IVF medium previously equilibrated under 5% CO_2_ and cultured for 17 h until fixation as above described (total number of oocytes examined 2,747).

Parameters evaluated were: penetration rate (number of oocytes penetrated/total inseminated), monospermy rate (number of oocytes containing only one sperm head-male pronucleus/total fertilized) and the ability of oocytes to sustain male pronucleus formation.

Degenerated and immature oocytes were not counted.

(*b*)* developmental competence of embryos after 7 days of in vitro culture.*

Based on the results obtained from in vitro fertilization, a set of experiments was carried out to evaluate the effect of Gly and R exposure during IVM on embryonic development of oocytes after IVF. At the end of maturation period in presence of different concentrations of Gly (0, 200 and 360 µg/mL) or R at the same Gly-equivalent doses, oocytes were coincubated with frozen-thawed spermatozoa for 1 h as described above, washed twice in IVF medium and incubated 3 h in the same medium. Then presumptive zygotes were washed twice in NCSU-23^68^ and cultured in 500 µL of the same medium. On Day 5 post-fertilization, 250 µL of the medium were replaced with fresh pre-equilibrated NCSU-23 containing 20% (v/v) FCS to reach a final FCS concentration of 10% (v/v). At Day 7 post-fertilization, percent of blastocysts and number of blastocyst nuclei were determined by fixing and staining embryos as above described for oocytes (total number of fertilized oocytes 2,634). Embryos with at least 20 blastomeres and a clearly visible blastocoel were considered as blastocysts.

### Evaluation of cumulus cell steroidogenesis

IVM media of both the first and the second day of culture of COCs in presence of Gly (0, 5, 10, 100, 200 and 360 µg/mL) or R at the same Gly-equivalent doses, were collected, centrifuged at 900×*g* for 5 min and the supernatants were stored at − 20 °C until assayed for progesterone (P4) and estradiol-17β (E2) by validated radioimmunoassays^52^. At the end of the maturation period, cumulus cells were counted using a Thoma’s hemocytometer, after being freed from matured oocytes by gentle repeated pipetting. For P4, the intra- and interassay coefficients of variation were 6.8% and 10.1%, respectively; assay sensitivity was 4.4 pg/tube. The intra- and interassay coefficients of variation for E2 were 5.4% and 10.5%, respectively; assay sensitivity was 1.7 pg/tube. Steroid concentrations are expressed as ng/10^6^ cells.

### Detection of GSH and ROS levels

Intracellular GSH and ROS levels of oocytes at the end of maturation period in presence of Gly (0, 100, 200 and 360 µg/mL) or R at the same Gly-equivalent doses, were determined using 4‑chloromethyl‑6.8‑difluoro‑7‑hydroxycoumarin (CellTracker Blue; CMF2HC; Invitrogen, Italy) or 2′,7′‑dichlorodihydrofluorescein diacetate (H2DCFDA; Invitrogen) respectively as previously described^36^. From each treatment group, oocytes were incubated in the dark for 30 min at 39 °C in PBS/0.1% (wt/vol) PVA supplemented with 10 μM H2DCFDA or 10 μM CellTracker Blue. Following incubation, the oocytes were washed in PBS/0.1% (wt/vol) PVA, placed into 10‑μL droplets, and fluorescence was evaluated under a Nikon Eclipse E 600 epifluorescence microscope (Nikon Europe BV, Badhoeverdop, The Netherlands). The fluorescence images were analysed with Image J software (public domain). Relative oocyte fluorescence was measured by normalizing the oocyte fluorescence with the background and with each oocyte area. Five independent experiments were performed (GSH samples, n = 747 oocytes; ROS samples, n = 804 oocytes).

### Statistical analyses

Statistical analyses were performed using R (version 3.4.0)^72^. Values are expressed as mean ± standard deviation (SD) and level of significance was at p < 0.05.

Data on nuclear maturation, IVF trials, blastocyst formation and cumulus cell steroidogenesis were analysed using a general linear model with binomial distribution and a Tukey post-hoc test was subsequently run to determine differences between treatments.

Data on blastomere number were analysed using a Poisson distribution and a Tukey post-hoc test was subsequently run to determine differences between treatments.

Data on GSH and ROS intracellular levels, after being tested for normality and homogeneity of variances through Shapiro–Wilk test, were analysed using Non-parametric Kruskal–Wallis Test and Wilcoxon test was subsequently used to assess differences between treatments.

## Data Availability

The datasets generated during and/or analysed during the current study are available from the corresponding author on reasonable request.
